# Accurate chromosome segregation by probabilistic self-organisation

**DOI:** 10.1186/s12915-015-0172-y

**Published:** 2015-08-12

**Authors:** Yasushi Saka, Claudiu V. Giuraniuc, Hiroyuki Ohkura

**Affiliations:** Institute of Medical Sciences, School of Medical Sciences, University of Aberdeen, Foresterhill, Aberdeen, AB25 2ZD UK; Wellcome Trust Centre for Cell Biology, University of Edinburgh, Michael Swann Building, Max Born Crescent, Edinburgh, EH9 3BF UK

**Keywords:** Chromosome segregation, Kinetochore, Microtubule, Mitosis, Meiosis, Markov chain, Self-organisation

## Abstract

**Background:**

For faithful chromosome segregation during cell division, correct attachments must be established between sister chromosomes and microtubules from opposite spindle poles through kinetochores (chromosome bi-orientation). Incorrect attachments of kinetochore microtubules (kMTs) lead to chromosome mis-segregation and aneuploidy, which is often associated with developmental abnormalities such as Down syndrome and diseases including cancer. The interaction between kinetochores and microtubules is highly dynamic with frequent attachments and detachments. However, it remains unclear how chromosome bi-orientation is achieved with such accuracy in such a dynamic process.

**Results:**

To gain new insight into this essential process, we have developed a simple mathematical model of kinetochore–microtubule interactions during cell division in general, i.e. both mitosis and meiosis. Firstly, the model reveals that the balance between attachment and detachment probabilities of kMTs is crucial for correct chromosome bi-orientation. With the right balance, incorrect attachments are resolved spontaneously into correct bi-oriented conformations while an imbalance leads to persistent errors. In addition, the model explains why errors are more commonly found in the first meiotic division (meiosis I) than in mitosis and how a faulty conformation can evade the spindle assembly checkpoint, which may lead to a chromosome loss.

**Conclusions:**

The proposed model, despite its simplicity, helps us understand one of the primary causes of chromosomal instability—aberrant kinetochore–microtubule interactions. The model reveals that chromosome bi-orientation is a probabilistic self-organisation, rather than a sophisticated process of error detection and correction.

**Electronic supplementary material:**

The online version of this article (doi:10.1186/s12915-015-0172-y) contains supplementary material, which is available to authorized users.

## Background

Accurate segregation of chromosomes during cell division is fundamental to life. Errors in this process result in cell death or aneuploidy. Chromosome segregation is usually very accurate. However, mis-segregation occurs at a much higher frequency in cancer cells and oocytes, which is a contributing factor to cancer progression [1] and also a major cause of infertility, miscarriages and birth defects such as Down syndrome [2].

The key event for chromosome segregation is the establishment of chromosome bi-orientation, in which sister chromatids in mitosis or homologous chromosomes in meiosis, attach to the microtubules from opposite spindle poles by kinetochores [3]. Each kinetochore consists of more than 100 different proteins assembled on each centromeric DNA sequence, many of which are involved in the interaction with microtubules [4]. Chromosome bi-orientation is a very dynamic process with frequent attachments and detachments of microtubules [5–8].

For proper segregation of chromosomes, all kinetochores need to attach to spindle microtubules while erroneous attachments must be eliminated before the onset of anaphase. It is known that attachment errors are more frequent in meiosis I (especially in oocytes) than in mitosis [2, 5–7]. Yet it is not understood why this is so. Unattached kinetochores act as signal generators for the spindle assembly checkpoint, which delays chromosome segregation until proper bi-orientation is established for all chromosomes [9]. It remains unclear, however, whether improperly attached kinetochore microtubules (kMTs) are also detected and corrected by the spindle assembly checkpoint or by an independent mechanism [10].

The precise mechanism of chromosome bi-orientation has been under intense investigations. However, it is not yet possible to observe the dynamics of individual microtubules in vivo in real time. Mathematical modelling provides a powerful means to study the chromosome bi-orientation process. Since the discovery of the dynamic instability of microtubules [11], a number of theoretical analyses have provided important insights into the interaction between microtubules and kinetochores (for example, [12, 13]). The so-called search-and-capture model explains how dynamically unstable microtubules capture chromosomes [14–17].

However, the original search-and-capture model did not concern events after capture, in particular, erroneous attachments of kMTs and their correction. To address this, Paul et al. put forward a modified search-and-capture model with explicit correction mechanisms [18]. Gay et al. proposed a stochastic model of kinetochore–microtubule attachments in fission yeast mitosis, which reproduced correct chromosome bi-orientation and segregation in simulations [19]. In addition to the kinetochore–microtubule interaction, Silkworth et al. showed that timing of centrosome separation also plays a crucial role for accurate chromosome segregation [20]; using experimental and computational approaches, they demonstrated that cells with incomplete spindle pole separation have a higher rate of kMT attachment errors than those with complete centrosome separation. Yet, the question remains unanswered as to how the cell can discriminate between correct and incorrect kMT attachments as their models assumed an explicit bias based on the discrimination of correct versus incorrect connections.

A major impediment to understanding fully the mechanism of chromosome bi-orientation is the lack of a universal theoretical framework that covers the chromosome bi-orientation process during eukaryotic cell divisions in general, including both mitosis and meiosis. Here we present such a universal model of chromosome bi-orientation, which is simple yet applicable to any eukaryotic cell division. Firstly, the model reveals that the balance between attachment and detachment probabilities of kMTs is crucial for correct chromosome bi-orientation. With the right balance, incorrect attachments are resolved spontaneously into correct bi-oriented conformations while an imbalance leads to persistent errors. Therefore, the superficially complex process, chromosome bi-orientation, is in fact a probabilistic self-organisation. It implies that the cell does not need to discriminate between correct and incorrect kMT attachments. Moreover, the model explains why errors are more frequent in meiosis I than in mitosis and how a faulty conformation can evade the spindle assembly checkpoint by a gradual increase of the number of kMTs. Despite its simplicity, the model is consistent with a number of experimental observations and provides theoretical insights into the origins of chromosomal instability and aneuploidy.

## Results and discussion

### A probabilistic model of kinetochore–microtubule interaction

**Fig. 1:**
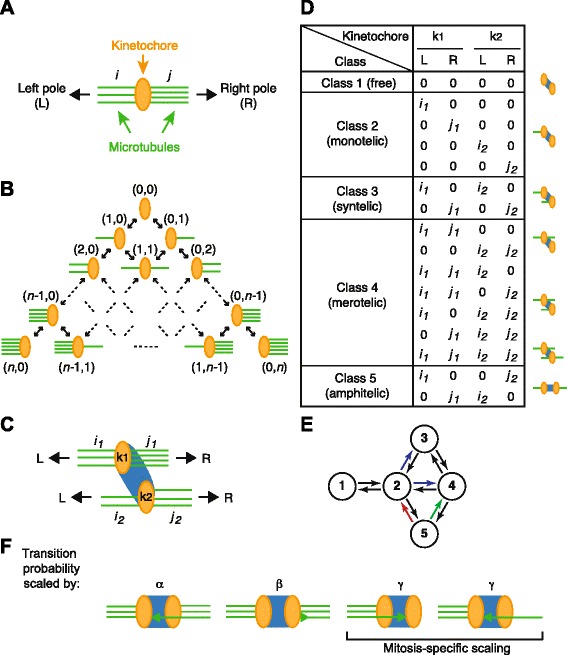
A discrete-time Markov chain model of kMT dynamics. **a** Schematic diagram of the interaction between a kinetochore (*orange*) and microtubules (*green*) from either left (*L*) or right (*R*) pole. *i* and *j* indicate the number of kMTs. **b** Kinetochore–microtubule interactions as a Markov chain. The maximal number of kMTs per kinetochore is *n*. **c** Schematic diagram of kMT dynamics during cell division. A pair of kinetochores (*k*_1_ and *k*_2_) are connected by bivalent chromatids in meiosis I or centromere chromatins (*blue*). **d** States of the kinetochore–microtubule complex are defined with *r*_*n*_(*i*_1_,*j*_1_,*i*_2_,*j*_2_). Every state can be classified into one of five classes in the table. Schematic diagrams of each class are shown on the right. **e** Transition diagram among classes. A subset of states in the Markov chain categorised in (**d**) can move from one class to another according to this diagram. To increase the probability of class 5 states, transitions out of class 5 (*red and green arrows*) must be reduced, the probabilities of which are scaled with parameters *α* (for the *green arrow*) and *β* (for the *red arrow*) in the model. In mitosis, transitions from class 2 to class 3 or 4 are scaled with *γ* (*blue arrows*). **f** Schematic diagram of the scaling by parameters *α*, *β* and *γ*. Probabilities of state transition by attachment or detachment (*arrowheads*) are scaled by the indicated parameters

A single kinetochore can bind randomly to microtubules from either left or right pole (Fig. 1a). We assume a single kinetochore can accommodate up to *n* microtubules. The process of microtubule attachment/detachment can be represented as a discrete-time Markov chain [21] (Fig. 1b and Additional file 1: Figure S1).

Each pair of sister chromatids in mitosis has two kinetochores (*k*_1_ and *k*_2_ in Fig. 1c). In meiosis I, a pair of sister kinetochores are physically connected side-by-side and act as one [22, 23]. Therefore, in our model, a bivalent (a pair of homologous chromosomes connected by chiasma) also has two kinetochores in meiosis I. We assume these two kinetochores interact with microtubules independently. Hence, the state of the kinetochores is represented as *r*_*n*_(*i*_1_,*j*_1_,*i*_2_,*j*_2_), which can be classified into one of five classes according to the pattern of microtubule attachments (Fig. 1d). State transitions occur in a stereotypical manner among these classes irrespective of the value of *n*≥2 (Fig. 1e and Additional file 1: Figure S2E; refer to Table 1 for a summary of the parameters herein). Notably, the only possible transitions out of class 5 (amphitelic, i.e. correct conformation) is to class 2 (monotelic) or 4 (merotelic) (red and green arrows in Fig. 1e). Note also that this transition scheme is similar to the kinetic error correction model (a deterministic ordinary differential equation model) proposed by Mogilner and Craig [24]; their scheme is a limiting case—only two kMT attachments per kinetochore are allowed and transitions out of amphitelic states are prohibited.

**Table 1:** Model parameters

	Parameter for	Range of values	Biological meaning
*n*	Maximal number of kMTs	2≤*n*	Maximal number of kMTs that can be accommodated on a single
	per kinetochore		kinetochore. *n* is proportional to the size of a kinetochore.
*p*	Association probability	0≤*p*≤1/4	2×*p* is the association probability of a single microtubule to a free
			kinetochore in each discrete time step. Upper limit of *p* is 1/4
			because total probability ≤1.
*q*	Dissociation probability	0≤*q*≤1/2*n*	Dissociation probability of a single kMT in each discrete time step.
*α*	Scaling factor of *p*	0≤*α*≤1	Scaling applies to transitions from amphitelic (class 5) to merotelic
			(class 4) states; reflecting the physical constraint imposed in
			amphitelic states (meiosis I) or the back-to-back position of sister
			kinetochores (mitosis). *α*=0 in mitosis for simplicity.
*β*	Scaling factor of *q*	0≤*β*≤1	Scaling applies to transitions in/from amphitelic states (class 5);
			reflecting the kMT stabilisation by tension.
*γ*	Scaling factor of *p*	0≤*γ*≤1	Scaling applies to transitions from monotelic (class 2) to syntelic
			(class 3) or merotelic (class 4) states in mitosis; reflecting the
			biased orientation of sister kinetochores in monotelic states.

We assume the association probability is proportional to the available surface area of the kinetochore while the dissociation probability is independent, as illustrated below:

(1) $$\begin{array}{@{}rcl@{}} r_{n}(i_{1},j_{1},i_{2},j_{2}) &\stackrel{\frac{n-i_{1}-j_{1}}{n} p }{\longrightarrow} & r_{n}(i_{1}+1,j_{1},i_{2},j_{2}),  \end{array} $$

(2) $$\begin{array}{@{}rcl@{}} r_{n}(i_{1},j_{1},i_{2},j_{2}) & \stackrel{i_{1} q \;}{\longrightarrow} & r_{n}(i_{1}-1,j_{1},i_{2},j_{2}),  \end{array} $$

where 0≤*p*≤1/4 and 0≤*q*≤1/2*n*. 2×*p* is the association probability of a single microtubule to a free kinetochore; *q* is the dissociation probability of a single kMT.

Experimental evidence strongly suggests that tension stabilises the spindle attachment to the kinetochores in amphitelic states (class 5) [25–27]. The stabilisation by tension is brought about by suppression of Aurora B kinase activity towards kinetochore substrates [27–30] as well as by mechanical catch-bonds [31, 32]. We model this stabilisation by scaling the transition probabilities of states in class 5 by detachment with the parameter 0≤*β*≤1 (Fig. 1f). This rule also reduces the probability of transitions from class 5 to class 2 states (Fig. 1e, red arrow). Similarly, the probability of class 5 (amphitelic) to class 4 (merotelic) transitions, which occur by attachment of a microtubule but not by detachment (Fig. 1e, green arrow), scales with 0≤*α*≤1 (Fig. 1f). This is due to the physical constraint imposed in amphitelic states in meiosis I [6, 7] or the kinetochore geometry (back-to-back position of sister kinetochores) in mitosis [3]. In mitosis, *α*=0 for simplicity. For mitosis we introduce an additional parameter 0≤*γ*≤1 to scale the transition probabilities from class 2 (monotelic) to class 3 (syntelic) or 4 (merotelic) (Fig. 1e blue arrows). This is because the biased orientation of sister kinetochores hinders those transitions (Fig. 1f). Note that when *α*=*β*=0, transitions out of class 5 are effectively blocked; hence, this Markov process always ends up in class 5. For additional details of the model, see Additional file 1. This simple model, which has only six parameters and is exactly solvable, provides a number of analytical insights into how correct chromosome bi-orientation is achieved.

### Dynamics of chromosome bi-orientation process

**Fig. 2:**
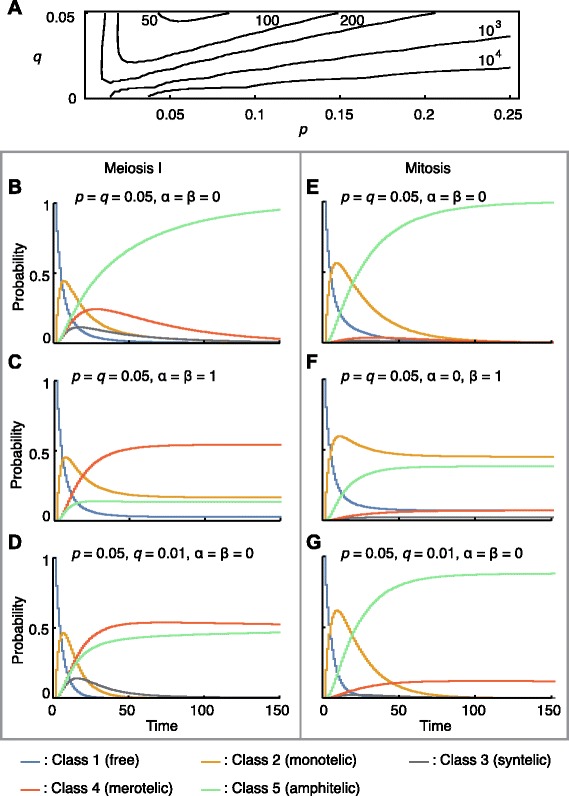
Dynamics of kinetochore–microtubule interaction. **a** Contour plot of mean first passage time to class 5 starting from class 1 in meiosis I. **b**–**g** Probabilities of each class over time for meiosis I (**b**–**d**) and mitosis (**e**–**g**). *n*=10 for all panels. *γ*=1 for meiosis I and *γ*=0.1 for mitosis. Other parameters are as indicated for each panel. **b**, **e** An ideal condition. The probability of class 5 approaches 1. **c**, **f** A random condition with no bias towards class 5. The probability of class 4 (merotelic) becomes predominant in meiosis I (**c**) while class 2 (monotelic) is as prevalent as class 5 (amphitelic) in mitosis (**f**). Note that classes 3 and 5 have identical probabilities by symmetry in (**c**). **d**, **g** A condition in which the *q*/*p* ratio is low. Class 4 persists both in meiosis I and in mitosis

The model predicts how long it takes to reach class 5 (amphitelic) from class 1 (free), i.e. the mean first passage time [33] (see Additional file 1). For a given value of *q*, the mean first passage time (which is independent of *α* and *β* because they only affect transitions out of class 5) is shortest when *p* is roughly equal to *q* (Fig. 2a and Additional file 1: Figure S3A–D). Thus, the relative dissociation rate (*q*/*p* ratio) of kMTs needs to be balanced for efficient chromosome bi-orientation.

The model also predicts the dynamics of the system (Fig. 2b–d for meiosis I and e–g for mitosis). Note that the *q*/*p* ratio dictates the dynamics of the Markov chain (Additional file 1: Figure S5). For both mitosis and meiosis in an ideal condition (*p*=*q*=0.05,*α*=*β*=0; Fig. 2b, e), the probability of class 5 steadily increases, asymptotically reaching 1. Notably, in meiosis I, class 4 (merotelic), and class 3 (syntelic) to a lesser extent, become transiently prominent (Fig. 2b). Merotelic attachments are indeed frequently observed in the prophase to prometaphase of meiosis I in mouse oocytes [7]. By contrast, in mitosis, class 2 (monotelic) becomes predominant before being replaced by class 5, although minor fractions of classes 3 and 4 also appear briefly (Fig. 2e). Together, this explains why meiosis I is more error-prone than mitosis; it is attributed to the parameter *γ*—the back-to-back conformation of sister kinetochores, which biases the kinetochore orientation.

If there is no bias in meiosis I (random condition; *α*=*β*=1; Fig. 2c, see also Additional file 1: Figure S4), the probability of class 5 stays low while that of class 4 (merotelic) reaches nearly 1/2 at steady states. This is because class 4 is by far the largest among the five classes (Additional file 1: Figure S2A, B). In mitosis, when the spindle tension is lacking (*β*=1; Fig. 2f), the model predicts a high probability of errors, mainly monotelic (class 2) states, as well as the correct amphitelic ones (class 5) at steady states. When kinetochore–microtubule attachment is stabilised by reducing *q*, merotelic errors (class 4) persist in both meiosis and mitosis (Fig. 2d, g). Class 5 will eventually replace class 4 but only very slowly; in meiosis I with *p*=0.05,*α*=*β*=0, the mean first passage times to class 5 are ∼1631 for *q*=0.01 versus ∼47 for *q*=0.05.

A number of studies have demonstrated that experimental manipulations of kinetochore–microtubule interactions lead to accumulation of incorrect spindle attachments (classes 1–4) and aneuploidy [8]. Lack of tension (i.e. *β*=1) makes amphitelic states (class 5) unstable [25–27]. Conversely, inhibition or depletion of aurora B kinase, which over-stabilises kMT attachments (by reducing *q*), causes errors in chromosome alignment and segregation [7, 27, 30, 34, 35]. These observations are consistent with our model predictions in which imbalance of the *q*/*p* ratio causes persistent errors in kMT attachments (Fig. 2).

### Probability distribution of the number of kMTs over time

**Fig. 3:**
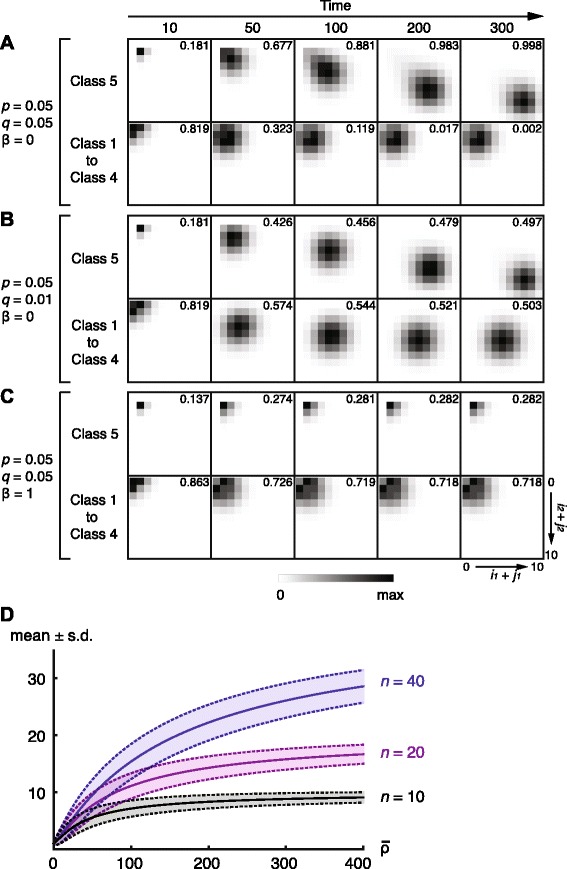
Probability distribution of the number of kMTs over time. **a**–**c** Probability density plots of the number of kMTs in meiosis I in 2D (*i*_1_+*j*_1_ vs. *i*_2_+*j*_2_; see Fig. 1 c) at the indicated time points. Parameters are indicated on the left. *α*=0, *n*=10 for all panels. Probabilities are decomposed into class 5 and the rest (class 1 to 4) at each time point. Total probabilities are indicated on each panel. The densities are scaled from 0 to the maximal for each panel. **d** Mean number of microtubules (± standard deviation) attached to a kinetochore derived by the approximation formulae (Eq. (3) and Additional file 1: Eq. (10)). Plots for *n*=10, 20 and 40 are shown. For details, see Additional file 1

Next, we calculated the probability distribution of the number of kMTs over time in different conditions (Fig. 3a–c and Additional file 1: Figure S6 for meiosis I; Additional file 1: Figure S7 for mitosis). We found qualitatively similar kMT distributions in mitosis and meiosis I, except the difference in the predicted phenotype in various conditions (Fig. 2b–g). The model predicts that in normal conditions (*p*=*q*=0.05,*α*=*β*=0) the number of kMTs increases steadily in class 5 while it remains low in the other classes as their total probability diminishes (Fig. 3a and Additional file 1: Figure S7A). This is in agreement with experimental evidence suggesting the gradual increase of kMTs during the prometaphase to metaphase in mitosis [36] and in meiosis I [26]. With smaller *q*, the number of kMTs increases not only in class 5 but also in class 4 (Fig. 3b and Additional file 1: Figure S7B). This explains why errors persist in this condition. Note that when *β*=0, the number of kMTs approaches *n*. An increasing number of kMTs may also switch off the spindle assembly checkpoint in merotelic states (class 4) over time.

These model predictions on the probability distribution of the number of kMTs have an important implication in the regulation of the spindle assembly checkpoint. Experimental evidence suggests that intrakinetochore stretching (or kinetochore deformation), which is brought about by kMT attachments, has a role in relieving the spindle assembly checkpoint [37–39]. Therefore, the predicted gradual increase of kMTs in amphitelic states (class 5) (Fig. 3a and Additional file 1: Figure S7A) may switch off the spindle assembly checkpoint progressively. The same argument applies to merotelic states (class 4), the probability of which increases when the *q*/*p* ratio is small (Fig. 2d, g); stabilisation of kMTs (Fig. 3b and Additional file 1: Figure S7B) may also inactivate the spindle assembly checkpoint in merotelic states over time. This explains why merotelic orientation evades the spindle assembly checkpoint [40], leading to aneuploidy. Intrakinetochore stretching by kMT attachment, however, does not allow the cell to discriminate between correct (amphitelic; class 5) versus incorrect (non-amphitelic; classes 1–4) kMT attachments [10]—the cell does not need to do so because chromosome bi-orientation occurs by probabilistic self-organisation as our model indicates.

We also examined how kMT number changes in amphitelic states under low spindle tension (*β*=1; Fig. 3c and Additional file 1: Figure S7C). Regardless of the classes, the distribution of kMT number remains low, which makes the transition of the process from one class to another more frequent. Similar probability distributions of kMT number in meiosis I were obtained when *α*=*β*=1 (Additional file 1: Figure S6A) and *α*=1,*β*=0 (Additional file 1: Figure S6B).

The exact probability distribution of kMT number at steady states can be derived in the special case when *α*=*β*=*γ*=1: its mean is $\bar {N}=n\rho /(n+\rho)$ where *ρ*=2*p*/*q* ($\bar {N}=5/3$ for *p*=*q*,*n*=10). We also obtained an analytical approximation of the kMT number distribution in class 5 when *α*=0:

(3) $$ \bar{N}_{5}=\frac{\bar{\rho} \left(\frac{\bar{\rho}}{n}+2\right)^{n-1}} {\left(\frac{\bar{\rho}}{n}+2\right)^{n}-2^{n}},  $$

where $\bar {\rho }=\rho /\beta =2p/(\beta q)$ (Fig. 3d and Additional file 1: Figure S8A, B). This formula is valid for both mitosis and meiosis and provides an analytical explanation as to how tension (*β*) alters the stability of kMTs by modulating the *q*/*p* ratio.

### Dynamics of multiple chromosomes

The above results concern the behaviour of a single pair of homologous chromosomes. It is natural to ask how multiple pairs in the cell are bi-oriented simultaneously—we call this event synchrony to distinguish it from the onset of anaphase. We assumed the system consists of *k* independent Markov processes. Let *θ*_*t*_ be the probability of a process being in class 5 (amphitelic) at time *T*=*t*, then the probability of synchrony at *T*=*t* is $\theta _{t}^{k}$ (see Additional file 1).

**Fig. 4:**
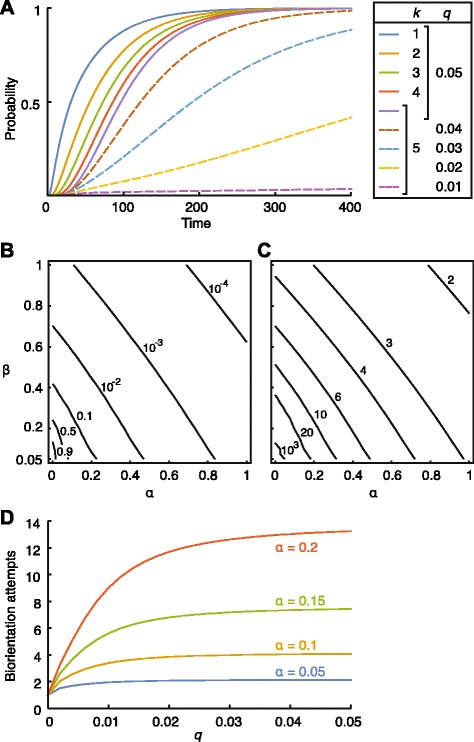
Dynamics of multiple chromosomes in meiosis I. **a** Probabilities of synchrony over time. *k*=number of chromosomes; *p*=0.05, *α*=*β*=0. **b** Contour plot of probability of synchrony at steady states. **c** Contour plot of half-life of synchrony at steady states. In (**b**) and (**c**), *p*=*q*=0.05 and *k*=5. **d** Number of bi-orientation attempts before absorption. *p*=0.05, *β*=0 and *n*=10 for all panels

The timing of synchrony delays as *k* increases (Fig. 4a and Additional file 1: Figure S3E, solid lines). If the balance of the *q*/*p* ratio is broken by reducing *q* (Fig. 4a and Additional file 1: Figure S3E, dashed lines), the timing of synchrony is delayed further (see also Additional file 1: Figure S9). The probabilities of synchrony, however, eventually approach 1 in all of these conditions with *β*=0. This implies that delaying the onset of anaphase could reduce the chromosome mal-orientation and mis-segregation. Consistently, Cimini et al. showed that prolonging the metaphase significantly reduced the number of lagging chromosomes in the anaphase (indicating incorrect kMT attachments) in mitosis [41].

We next examined the contribution of *α* and *β* to the establishment of synchrony. Figure 4b shows the steady-state probability of synchrony in meiosis I as a contour plot. It indicates that, to achieve synchrony reliably at steady states, *α* and *β* have to be relatively small. It is conceivable that, to progress into anaphase, synchrony has to be maintained for a sufficient time to relieve the spindle assembly checkpoint [10]. Figure 4c depicts the half-life of synchrony in meiosis I as a contour plot (see also Additional file 1: Figure S3F for mitosis). The half-life increases steeply for small values of *α* and *β*. These data suggest that *α* and *β* need to be tightly regulated for efficient chromosome bi-orientation and segregation accuracy.

### Error correction of kMT attachments in meiosis I

Finally, we asked how many rounds of error correction of kMT attachments occur in meiosis I before the establishment of correct bi-orientation (see Additional file 1 for methods). We calculated the number of bi-orientation attempts per bivalent, i.e. the mean number of transitions from class 2 or 4 to class 5 before the kinetochore is fully occupied (*r*_*n*_(*n*,0,0,*n*) and *r*_*n*_(0,*n*,*n*,0) when *β* = 0) (Fig. 4d). It suggests that the larger *α* is, the more bi-orientation attempts are needed. We also found the number of bi-orientation attempts decreases as *q* (detachment probability) reduces (Fig. 4d, see also Additional file 1: Figure S10). Consistent with this, Kitajima et al. observed the number of attempts reduced from ∼3 in untreated mouse oocytes to just one on average in those treated with hesperadin, an Aurora B kinase inhibitor [7].

## Conclusions

Our simple discrete-time Markov chain model captures the prominent features of the chromosome bi-orientation process. It provides a unified account of two modes of divisions, mitosis and meiosis I, under a single theoretical framework. The model reveals where the differences in the bi-orientation process come from and it explains why errors are very frequent in the first meiotic division, which are major causes of infertility, miscarriages and birth defects in humans.

One of our key findings in this study is that the system dynamics (including the type and frequency of transient kMT attachment errors) is dictated by the *q*/*p* ratio (relative detachment rate) of kMTs. An imbalance of the *q*/*p* ratio causes persistent attachment errors leading to chromosome mis-segregations. The gradual increase of kMTs may help turn off the spindle assembly checkpoint in normal conditions but can promote a faulty conformation (merotelic attachments) to evade the checkpoint.

In summary, our study revealed that chromosome bi-orientation is a probabilistic self-organisation, rather than a sophisticated process of error detection and correction. Although our model omits many potentially important factors for chromosome bi-orientation, such as the spatial arrangement of centrosomes, it allowed us to examine analytically all possible outcomes with different parameters (i.e. the whole parameter space), revealing what is fundamental for accurate chromosome segregation. The proposed model, which is based on a firm mathematical foundation, gives valuable insights that help us understand one of the primary causes of chromosomal instability—aberrant kMT dynamics.

## Methods

The model and its analysis are explained in detail in Additional file 1. The analysis of discrete-time Markov chains was performed according to [21, 33, 42]. We used Mathematica®; (version 10, Wolfram Research) to implement and analyse the model, with a standard laptop (or desktop) computer. The Mathematica codes used in this study are provided in Additional file 2.

## Additional files

Additional file 1
**Supplementary Information.** Details of the model construction and analysis and **Figures S1–S10**. (PDF 930 kb)

Additional file 2
**Mathematica codes.** The Mathematica codes used in this study. To read the file, Wolfram CDF player (available free from https://www.wolfram.com/cdf-player/) or Mathematica (Wolfram Research) is required. (ZIP 62 kb)

## Abbreviation

kMT: kinetochore microtubule
