# Supplementary material for: Accurate chromosome segregation by probabilistic self-organisation
Source: BMC Biol. 2015 Aug 12;13:65. doi: 10.1186/s12915-015-0172-y (PMC4533937; doi:10.1186/s12915-015-0172-y)
Supplement: Additional file 1 — Supplementary Information. Details of the model construction and analysis and Figures S1–S10. (PDF 930 kb) [file 12915_2015_172_MOESM1_ESM.pdf]

Accurate chromosome segregation by probabilistic self-organization

Yasushi Saka [1], Claudiu V Giuraniuc [1] and Hiroyuki Ohkura [2]

[1] Institute of Medical Sciences, School of Medical Sciences, University of Aberdeen, Foresterhill, AB25 2ZD Aberdeen, UK
[2] Wellcome Trust Centre for Cell Biology, University of Edinburgh, Michael Swann Building, Max Born Crescent, EH9 3BF, Edinburgh, UK

Additional file 1

1 A basic Markov chain model of kinetochore-microtubule interactions (Model I)

The interaction of a single kinetochore with microtubules is modeled as a birth/death (discrete-time) Markov process. First, we consider a kinetochore that can bind up to n microtubules. The possible states are $M = \{0, 1, 2, ..., n\}$. Transition probability from state i to j is $p_{i,j} = P\left(X_{t+1} = j \,|X_t = i\right),\ i, j \in M$, where X_t is the state at time t. As stated in the main text, we assume the association probability is proportional to the surface area of a kinetochore available for microtubule attachment. Therefore, the association (birth) probability is $p_{k,k+1} = (n-k)\,b/n,\ 0 \le k \le n-1$ where b is the association probability of a single microtubule to a free kinetochore. The dissociation (death) probability for state k is $p_{k,k-1} = k\,d,\ 1 \le k \le n$, because each microtubule bound to a kinetochore (k microtubules in total) has the same dissociation probability d. The self-transition probability is $p_{k,k} = 1 - (n-k)\,b/n - k\,d,\ 0 \le k \le n$. As an example, consider a kinetochore that can bind up to 2 microtubules. The possible states are $\{0, 1, 2\}$. The transition probability matrix is

$$R_3 = \begin{pmatrix} p_{0,0} & p_{0,1} & p_{0,2} \\ p_{1,0} & p_{1,1} & p_{1,2} \\ p_{2,0} & p_{2,1} & p_{2,2} \end{pmatrix} = \begin{pmatrix} 1-b & b & 0 \\ d & 1-\frac{1}{2}b-d & \frac{1}{2}b \\ 0 & 2d & 1-2d \end{pmatrix}.$$

Fig. S1A shows a diagram of this Markov chain. This model is a variation on the M/M/s queue [1]. The Markov chain consists of a single aperiodic recurrent class. Let u_k be the steady-state probabilities of state k and U be the row vector of $u_k, k = 0, 1, 2, ..., n$. Applying the steady-state convergence theorem, then $U = UR_n$. This is equivalent to a local balance equation: $(1 - k/n)\,b\,u_k = (k+1)d\,u_{k+1}$. Let $\rho = b/d$ then,

$$(1 - k/n)\rho u_k = (k+1)u_{k+1}, k = 0, 1, ..., n-1. \tag{4}$$

The normalization equation (the sum of all probabilities equals to 1) is

$$\sum_{k=0}^{n} u_k = 1. \tag{5}$$

Eqs. (4) and (5) yield a unique solution:

$$u_k = \binom{n}{k}(\rho/n)^k(1+\rho/n)^{-n},\ k = 0, 1, 2, ..., n, \tag{6}$$

where $\binom{n}{k}$ is the binomial coefficient ("n choose k"). When n is large, u_k approaches the Poisson distribution $e^{-\rho}\,\rho^k\big/k!$. The mean and variance of u_k, derived from Eq. (6), are $n\rho/(n+\rho)$ and $n^2\rho\,\big/(n+\rho)^2$, respectively. Fig. S1B shows an example of the probability distribution of u_k (the number of attached microtubules) for $n = 20$. As illustrated in this example, the stability of kinetochore-microtubule interaction can be controlled by ρ (i.e. d/b ratio) alone.

2 The extended model of kinetochore-microtubule interactions (Model II)

Now we consider the interaction of a kinetochore with bipolar spindles (Fig. 1B in the main text). There are $(n+1)(n+2)/2$ possible states, e.g. 6 possible states for $n = 2$. We assign a unique index number to each state denoted as $s_n(i, j)$:

$$s_n(i,j) \longmapsto i + 1 + \frac{(i+j+1)(i+j)}{2},\ 0 \le i + j \le n. \tag{7}$$

We use these indices to construct the probability transition matrix in *Mathematica* codes. Using the same argument for model I, the state transition probabilities are

$$\begin{aligned} s_n(i,j) &\xrightarrow{\frac{n-i-j}{n}p} s_n(i+1,j), \\ s_n(i,j) &\xrightarrow{\frac{n-i-j}{n}p} s_n(i,j+1), \\ s_n(i,j) &\xrightarrow{i\,q} s_n(i-1,j), \\ s_n(i,j) &\xrightarrow{j\,q} s_n(i,j-1), \end{aligned} \tag{8}$$

where p, q are parameters with $0 \le p \le 1/2,\ 0 \le q \le 1/n$. For example, the transition matrix P_n with $n = 2$ is

$$P_2 = \begin{pmatrix} 1-2p & p & p & 0 & 0 & 0 \\ q & 1-p-q & 0 & \frac{p}{2} & \frac{p}{2} & 0 \\ q & 0 & 1-p-q & 0 & \frac{p}{2} & \frac{p}{2} \\ 0 & 2q & 0 & 1-2q & 0 & 0 \\ 0 & q & q & 0 & 1-2q & 0 \\ 0 & 0 & 2q & 0 & 0 & 1-2q \end{pmatrix}.$$

Model II is fundamentally the same as Model I: b in Model I is equal to the combined probability of a microtubule binding to a free kinetochore ($= 2 \times p$) in Model II. Dissociation probability of a single kMT is the same ($d = q$). Hence $\rho = 2p/q$.

3 The model of kinetochore-microtubule interactions in meiosis and mitosis (Model III)

The model of kinetochore-microtubule interactions in meiosis and mitosis is built from Model II, which we call Model III. This model describes the state of a pair of kinetochores physically connected by a centromere chromatin (in mitosis and meiosis II) or a bivalent (in meiosis I) of homologous chromosomes, which is defined by $r_n\,(i_1, j_1, i_2, j_2)$. Note that $0 \le p \le 1/4$ and $0 \le q \le 1/2n$ in Model III because the total transition probability from a given state including self-transition is 1. Also note that there is no direct transition from class 1 (free) to class 5 (amphitelic, i.e. correct conformation).

As briefly mentioned in the main text, spindle tension stabilises the kMT attachments in amphitelic states (class 5), which is represented by the scaling with the parameter β. This applies to both mitosis and meiosis. The scaling with the parameter β is exemplified by

$$r_n(i_1,0,0,j_2) \xrightarrow{i_1\beta\,q} r_n(i_1-1,0,0,j_2).$$

This rule also reduces the probability of transitions from class 5 to class 2 states (red arrow in Fig. 1E; with $i_1 = 1$ in the above example).

The scaling of the probability of class 5 (amphitelic) to class 4 (merotelic) transitions with the parameter α is based on the experimental evidences. In amphitelic states in mitosis, the kinetochore geometry in mitotic chromosomes prevents each sister kinetochore from interacting with the microtubules from the opposite pole [2]. Therefore class 5 (amphitelic) to class 4 (merotelic) transitions are effectively eliminated in mitosis, i.e. $\alpha = 0$. In meiosis I, Nicklas suggested that the stability of amphitelic conformation is also gained by the aligned position

of kinetochores with the pole-to-pole axis, with each kinetochore pointing at a pole [3]. A recent study of meiosis I in mouse oocyte indeed revealed the restricted movement of kinetochores in amphitelic states (see supplemental movies in Kitajima et al [4]). The scaling with the parameter α is exemplified by

$$r_n(i_1, 0, 0, j_2) \xrightarrow{\frac{n-i_1}{n}\alpha\, p} r_n(i_1, 1, 0, j_2).$$

With a similar reason transitions from class 2 (monotelic) to class 3 (syntelic) or 4 (merotelic) are reduced in mitosis because the attached sister kinetochore are facing towards the pole from which the kMT emanates, while the other unattached sister kinetochore are facing the opposite pole. Thus, these transitions (blue arrows in Fig. 1E) are scaled by the parameter $0 \le \gamma \le 1$. For example,

$$\begin{array}{lcl} r_n(i_1, 0, 0, 0) & \xrightarrow{\gamma\, p} & r_n(i_1, 0, 1, 0), \\ r_n(i_1, 0, 0, 0) & \xrightarrow{\frac{n-i_1}{n}\gamma\, p} & r_n(i_1, 1, 0, 0). \end{array}$$

These scaling of the transitions by γ are unique to mitosis (and meiosis II); for meiosis I, $\gamma = 1$.

With sufficiently small α and β, class 5 becomes stable; when $\alpha = \beta = 0$, transitions out of class 5 are not possible. That means class 5 is an absorbing class in the Markov chain. This bias towards class 5 underpins the probabilistic self-organisation of the system. By contrast, when $\alpha = \beta = 1$ there is no bias towards class 5, that is, amphitelic states are unstable. Note that when $\alpha \ne 0, \beta = 0$, the process eventually ends up in either $r_n(n, 0, 0, n)$ or $r_n(0, n, n, 0)$, that is, the class 5 states with maximal number of kMTs.

4 Steady-state PMF (probability mass function) in Model II

To calculate the steady-state PMF in Model II, consider it as a process of choosing the number of microtubules per kinetochore and distributing them to left and right poles. Let $k(\le n)$ be the total number of microtubules attached to the kinetochore and $\phi_n(i, j)$ be the PMF for state $s_n(i, j)$, then $\sum_{i=0}^{k} \phi_n(i, j) = u_k,\ i + j = k$. $\phi_n(i, j)$ is derived by distributing u_k according to the binomial distribution:

$$\binom{i+j}{i}(1/2)^i(1/2)^j = \binom{k}{i}(1/2)^k,\ 0 \le i \le k.$$

Hence, using Eq. (6),

$$\begin{aligned} \phi_n(i, j) &= u_k \times \binom{k}{i}(1/2)^k \\ &= \left(1 + \frac{\rho}{n}\right)^{-n} \left(\frac{\rho}{2n}\right)^{i+j} \frac{n!}{i!j!(n-i-j)!}. \end{aligned} \quad (9)$$

Let $\Phi_n = (\phi_n(0,0), \phi_n(0,1), \phi_n(1,0), \phi_n(0,2), \phi_n(1,1), ..., \phi_n(n-1,1), \phi_n(n,0))$. Then, by applying Eq. (8) and (9), we find $\Phi_n.P_n = \Phi_n$, which is consistent with the equilibrium at steady states.

5 Size of the Markov chains

The size of a Markov chain in Model II (total number of states) corresponds to the maximum of the $s_n(i, j)$ indices according to Eq. (7), which is $(n + 1)(n + 2)/2$. The total number of states in the full model (Model III) is thus $(n + 1)^2\ (n + 2)^2/4$, which grows rapidly as n increases (Fig. S2A). Note that class 4 becomes predominant as the system size gets larger (Fig. S2B). Consequently, the number of possible state transitions also increases exponentially with the system size (Fig. S2C), which corresponds to the number of non-zero entries in the probability transition matrix.

6 First passage time to class 5

For a Markov chain of Model III, the mean first passage time f_i to class 5 from state i is obtained as the solution of linear equations [5]:

$$f_i = \begin{cases} 1 + \sum\limits_{j \notin \text{class } 5} p_{i,j} f_j, & i \notin \text{class } 5, \\ 0, & i \in \text{class } 5. \end{cases}$$

f_1 in Fig. 2A was calculated by incrementing p and q by 0.005 (50 $\times$ 10 points) and in Figs. S3A and S3D by 0.0001 (100 $\times$ 100 points). For a given value of q $(= 0.0005)$, the minimum f_1 plateaus as n grows (Fig. S3B), although the number of states and transitions increase rapidly (Figs. S2A and S2C). For meiosis I, the q/p ratio for the minimum f_1 approaches $\sim$1 as n increases (Fig. S3C). For mitosis, the optimal q/p ratio is somewhat skewed (Fig. S3D).

7 PMF time series of $r_n\,(i_1, j_1, i_2, j_2)$

It is straightforward to calculate the PMF of $r_n\,(i_1, j_1, i_2, j_2)$ at each time point from the transition probability matrix. We classified the PMFs according to Fig. 1D and calculated the sums for each class to obtain Fig. 2B to G. Fig. S4 shows the PMF time series in meiosis I with $\alpha = 0, \beta = 1$ (a) and $\alpha = 1, \beta = 0$ (b) ($n = 10, p = q = 0.05$). Class 4 is predominant in these condition as well.

The dynamics are qualitatively very similar with any n; this is mainly because the structure of the Markov chain remains the same as the size of the chain grows (Fig. 1B and S2E). We have used $n = 10$ for most of the analysis as a representative value. We extensively explored the dynamics with different values of n and found fundamentally no diffence in the behavior of the Markov chain by altering n (for example, $n = 10$ versus 15 in Figs. S5B, C, E and F).

8 Invariant dynamics of the Markov process with constant q/p ratio

As long as q/p ratio (relative kMT dissociation rate) remains the same, the steady-state probabilities of $r_n\,(i_1, j_1, i_2, j_2)$ stay the same for all p, q pairs. The PMF time series are also almost invariable (but with different time scales) as long as q/p ratio remains constant, illustrated by the examples shown in Fig. S5. Only the time scale changes, which is inversely proportional to $\sqrt{p\,q}$. Strictly speaking, although steady-state probabilities are identical, PMFs at any given moment are not exactly the same: these small differences come about by the assumption that only a single event happens in every state transition. Differences are small enough to be ignored when p and q are sufficiently small. From a biological perspective, the change in time scale with the same dynamics has a different meaning: the faster the association and dissociation of kMTs, the more efficient the chromosome biorientation. See also the section 'Biorientation attempts' below.

9 Probability distribution of kMTs at steady states in meiosis I.

Class 5

Steady state probability distributions of the number of kMTs in class 5 for $n = 10,\ p = 0.1,\ q = 0.05$ are shown in Fig. S8B as density plots. Total probability of class 5 is indicated for each panel. Gray scale is normalized to the total probability of class 5. When α and β are sufficiently small, class 5 (amphitelic) states are stable, i.e. the number of kMTs are close to the maximum. Otherwise, only a few microtubules on average are attached to each kinetochore.

Class 1 to 4

Steady state probability distributions of the number of kMTs in classes 1 to 4 for $n = 10,\ p = 0.1,\ q = 0.05$ are shown in Fig. S8C as density plots. Although the total probabilities are greatly affected by the parameters α

and β, the distribution of the number of kMTs of non-class 5 states barely changes. This is presumably because the size of the non-amphitelic classes (mainly class 4) in total is significantly larger than that of class 5 (Fig. S2B), buffering the influence of class 5. Thus, $\bar{N} = n\rho/(n+\rho)$ (the exact solution in the random condition $\alpha = \beta = \gamma = 1$) is also an approximate of the steady state distribution of kMTs in meiosis I for non-amphitelic states when $\alpha \neq 1, 0 < \beta \neq 1$.

10 Number of kMTs at steady states in class 5 — an analytical approximation

When $\alpha = 0$, the number of kMTs for states in class 5 can be estimated analytically without explicitly calculating the PMF, which is computationally expensive for large n. The reason why it is possible becomes apparent by looking at the Markov chain's structure and transitions — when $\alpha = 0$, transitions from class 5 to class 2 are still possible, but not to class 4 anymore. Note that for large n the number of transitions between class 5 and class 4 is far larger than the one between class 5 and class 2 (Fig. S2D).

Because of its limited communication with other classes when $\alpha = 0$, class 5 behaves as if it is a disjoint class at steady states. Its two sub-classes (e.g. left and right square grids in Fig. S2E) have the identical probability distribution by symmetry, which can be approximated by

$$\begin{aligned}\psi_n(i,j) &= \phi_n(i,0) \times \phi_n(0,j) \\ &= 2^{-(i+j)} u_i \, u_j \\ &= \binom{n}{i}\binom{n}{j}\left(\frac{\bar{\rho}}{2n}\right)^{i+j}\left(1+\frac{\bar{\rho}}{n}\right)^{-2n},\end{aligned}$$

where $1 \leq i,j \leq n$ and $\bar{\rho} = \rho/\beta = \frac{2p}{\beta q}$. We now compute the conditional expectation of the number of kMTs i (or j) given the state s is in class 5:

$$\begin{aligned}E(i|s \in \text{class } 5) &= \sum_{i=1}^{n}\sum_{j=1}^{n}\left(\psi_n(i,j) \times i\right) \Big/ \sum_{i=1}^{n}\sum_{j=1}^{n}\psi_n(i,j) \\ &= \bar{N}_5.\end{aligned}$$

After a lengthy algebra, it simplifies to Eq. (3) in the main text. Similarly,

$$E\left(i^2 \middle| s \in \text{class } 5\right) = \sum_{i=1}^{n}\sum_{j=1}^{n}\left(\psi_n(i,j) \times i^2\right) \Big/ \sum_{i=1}^{n}\sum_{j=1}^{n}\psi_n(i,j).$$

After another lengthy calculation, variance of i is reduced to:

$$\begin{aligned}\text{Var}(i|s \in \text{class } 5) &= E\left(i^2 \middle| s \in \text{class } 5\right) - \left(E(i|s \in \text{class } 5)\right)^2 \\ &= \frac{\bar{\rho}\left(2+\frac{\bar{\rho}}{n}\right)^{n-2}\left(2\left(2+\frac{\bar{\rho}}{n}\right)^{n} - 2^n\left(2+\bar{\rho}\right)\right)}{\left(\left(2+\frac{\bar{\rho}}{n}\right)^{n} - 2^n\right)^2}. \qquad (10)\end{aligned}$$

Eqs. (3) and (10) fit very well to the exact number of kMTs derived from the steady-state PMF (Fig. S8A). When n is small (e.g. $n = 4$), the approximation diverges a little from the exact values, but is still pretty good (not shown). The mean and variance approach n and 0, respectively, as $\bar{\rho} \to \infty$.

11 Probability of synchrony

Computing the probability of synchrony

We compute the probability of synchrony at time $T = t$, i.e. the probability that the process in every chain is in the same class (in particular class 5) at the same time. This is illustrated by an example below, which shows the state

(class) transtions of each process (Ch.1 to 4) from $T=0$ to 20. Synchrony in class 5 is highlighted in red, which occurs at $T=18$.

	0	1	2	3	4	5	6	7	8	9	10	11	12	13	14	15	16	17	18	19	20
Ch.1	1	2	1	1	1	1	1	1	2	3	4	4	4	4	4	2	2	2	5	5	5
Ch.2	1	1	1	1	1	1	1	1	2	4	4	4	4	4	4	4	5	5	5	5	5
Ch.3	1	2	1	1	1	1	1	2	3	3	2	5	5	5	5	5	5	5	5	5	5
Ch.4	1	2	3	3	2	2	2	3	4	4	4	4	5	5	5	5	5	5	5	5	5

Let $s[t]$ be the state of a single Markov process at time $T=t$, $a_j^{(t)}$ be the probability of $s[t]=j$ and k be the total number of chains. In the above example, $k=4$. Also let $P_s^{(t)}$ and $P_{as}^{(t)}$ be the probability of synchrony and asynchrony at $T=t$, respectively. Then,

$$\begin{aligned} P_s^{(t)} &= {\theta_t}^k, \\ P_{as}^{(t)} &= 1-P_s^{(t)}, \\ &= 1-{\theta_t}^k, \end{aligned} \tag{11}$$

where

$$\theta_t = P(s[t] \in \text{class } 5) = \sum_j a_j^{(t)},\ j \in \text{class } 5.$$

We used Eq. (11) for Figs. 4A and S3E. Now we consider the probability of synchrony attempts. For this, we need some events and their probabilities defined. The probability of biorientation attempts $P_+^{(t)}$ in a single process is

$$\begin{aligned} P_+^{(t)} &= P(s[t-1] \notin \text{class } 5 \wedge s[t] \in \text{class } 5) \\ &= \sum_{i,j} a_i^{(t-1)} p_{i,j},\ i \notin \text{class } 5,\ j \in \text{class } 5, \end{aligned}$$

where $p_{i,j}$ is the transition probability from state i to j. Likewise, the probability of biorientation loss $P_-^{(t)}$ is

$$P_-^{(t)} = \sum_{i,j} a_j^{(t-1)} p_{j,i},\ i \notin \text{class } 5,\ j \in \text{class } 5.$$

The probability of biorientation maintainance $P_0^{(t)}$ of a process is

$$\begin{aligned} P_0^{(t)} &= P(s[t-1] \in \text{class } 5 \wedge s[t] \in \text{class } 5) \\ &= \sum_{i,j} a_i^{(t-1)} p_{i,j},\ i \in \text{class } 5, j \in \text{class } 5. \end{aligned}$$

Let m be the number of Markov processes in class 5 at $T=t$. Note that a synchrony attempt occurs only when all m processes that are in class 5 stay in class 5 and the remaining $k-m$ processes undergo transition from non-class 5 to class 5 states. The probability of such a synchrony attempt, $P_{\text{as},s}^{(t)}$, is

$$\begin{aligned} P_{as,s}^{(t)} &= P(\text{asynchrony at } T=t-1 \wedge \text{synchrony at } T=t) \\ &= \sum_{m=0}^{k-1} \binom{k}{m} \left(P_+^{(t)}\right)^{k-m} \left(P_0^{(t)}\right)^m, \end{aligned}$$

where $\binom{k}{m}$ is the binomial coefficient. Note that Fig. S9A may help understand the following derivation of formulae.

The probability of synchrony maintenance $P_{s,s}^{(t)}$ is

$$\begin{aligned} P_{s,s}^{(t)} &= P(\text{synchrony at } T=t-1 \wedge \text{synchrony at } T=t) \\ &= P_s^{(t)} - P_{as,s}^{(t)}. \end{aligned}$$

Likewise, the probability of asynchrony maintenance $P_{as,as}^{(t)}$ is

$$\begin{aligned} P_{as,as}^{(t)} &= P(\text{asynchrony at } T = t-1 \wedge \text{asynchrony at } T = t) \\ &= P_{as}^{(t)} - P_{s,as}^{(t)}. \end{aligned}$$

The probability of synchrony loss $P_{s,as}^{(t)}$ is obtained by

$$\begin{aligned} P_{s,as}^{(t)} &= P(\text{synchrony at } T = t-1 \wedge \text{asynchrony at } T = t) \\ &= P_{s}^{(t-1)} - P_{s,s}^{(t)} \\ &= P_{s}^{(t-1)} - \left(P_{s}^{(t)} - P_{as,s}^{(t)}\right) \\ &= P_{as,s}^{(t)} - \left(P_{s}^{(t)} - P_{s}^{(t-1)}\right). \end{aligned} \tag{12}$$

$P_{s,as}^{(t)}$ can also be obtained by

$$P_{s,as}^{(t)} = \sum_{m=1}^{k} \binom{k}{m} \left(P_{-}^{(t)}\right)^{m} \left(P_{0}^{(t)}\right)^{k-m}.$$

At steady states, the conditional probability of synchrony loss given the present state is in synchrony is $P_{s,as}^{(\infty)}/P_{s}^{(\infty)}$, therefore the mean duration (half-life) of synchrony is $P_{s}^{(\infty)}/P_{s,as}^{(\infty)}$. Fig. 4C was derived by this formula.

Now we examine when the synchrony happens for the first time, i.e. the probability of the first synchrony at $T = t$, denoted by $P_{fs}^{(t)}$. With $\alpha = \beta = 0$, once a process is in class 5, it is trapped in the class (i.e. $P_{s,as}^{(t)} = 0$). Therefore $P_{fs}^{(t)} = P_{as,s}^{(t)}$. When α and β are small, the majority of synchrony attempts are for the first time; in addition, as k gets larger (number of processes, i.e. pairs of sister chromatids in mitosis or bivalents in meiosis I), synchrony becomes a rarer event. Thus, the probability of synchrony loss $P_{s,as}^{(t)}$ are small at any given moment for large k and small α and β. In such a condition, it is therefore possible to approximate $P_{fs}^{(t)}$ with $\tilde{P}_{fs}^{(t)}$:

$$\begin{aligned} \tilde{P}_{fs}^{(t)} &= P_{as}^{(0)} \\ &\quad \times \left(\prod_{\tau=1}^{t-1} P(\text{asynchrony at } T = \tau-1 \wedge \text{asynchrony at } T = \tau \,|\, \text{asynchrony at } T = \tau-1)\right) \\ &\quad \times P(\text{asynchrony at } T = t-1 \wedge \text{synchrony at } T = t \,|\, \text{asynchrony at } T = t-1) \\ &= \left(\prod_{\tau=1}^{t-1} \frac{P_{as,as}^{(\tau)}}{P_{as}^{(\tau-1)}}\right) \times \frac{P_{as,s}^{(t)}}{P_{as}^{(t-1)}},\ t = 2, 3, 4\ldots. \end{aligned}$$

It is apparent that for $\alpha = \beta = 0$ (therefore $P_{s,as}^{(t)} = 0$), $\tilde{P}_{fs}^{(t)} = P_{as,s}^{(t)}$. Fig. S9B shows an example of $\tilde{P}_{fs}^{(t)}$ together with Monte Carlo simulation results (probability in 5,000 simulations), demonstrating a good fit of the approximation to the simulation result.

Timing of first synchrony and q/p ratio

We asked how q/p ratio affects the timing of first synchrony. We also asked how efficiently synchrony can be achieved in a slightly compromized condition, i.e. $\alpha = \beta = 0.05$. Fig. S9C shows the probability of first synchrony in meiosis I at each time point with decreasing q value ($n = 10,\ p = 0.05$ and the number of bivalents $k = 5$). When $p = q = 0.05$, first synchrony happens most frequently around $T = 100$; By $T = 400$ synchrony takes place at least once in $\sim 99.7\%$ of cases (not shown). As q/p ratio declines, the timing of first synchrony spreads more and more over time, becoming unpredictable. Therefore, synchrony does happen relatively efficiently with the right q/p ratio even in a slightly compromized condition with $\alpha = \beta = 0.05$. For a fixed value of $p = 0.05$, the probability of synchrony at steady state (at any give moment) is 0.66 with $q = 0.05$, but only 0.017 when $q = 0.01$. For $k = 20$, the probability declines to 0.19 with $q = 0.05$ and 8.3×10^{-8} with $q = 0.01$. Thus, keeping the balance of q/pratio is all the more important for the cell with a large number of chromosomes. This principle applies to both mitosis and meiosis I.

12 Bi-orientation attempts

Probability of bi-orientation attempts

Probability of biorientation attempts at time $T = t$, μ_t, is

$$\mu_t = \sum_{i,j} a_i^{(t)} p_{i,j},\ i \notin \text{class } 5, j \in \text{class } 5,$$

where $a_i^{(t)}$ is the probability of the process in state i at time t and $p_{i,j}$ is the transition probability from state i to j. μ_t can also be interpreted as the mean number of attempts to biorientation at time t. Fig. S10A shows a plot of μ_t by this formula (analytical solution) and simulations (parameters: $n = 5, p = q = 0.01, \alpha = \beta = 0.1$; 10,000 simulations). Fig. S10B shows an example of probability time series of biorientation attempts, with $p = q = 0.05$ versus $p = q = 0.01$ ($n = 10$, $\alpha = \beta = 0.1$). With the same q/p ratio, their PMF time series are almost identical (not shown) if the time scale is adjusted. Because of this change of time scale, the probability (i.e. frequency) of biorientation attempts also changes. In this example, the probability at steady states (at any time point) decreases from ∼0.033 with $p = q = 0.05$ to ∼0.0066 with $p = q = 0.01$.

Mean number of biorientation attempts before absorption

The number of biorientation attempts before the onset of anaphase is equivalent in our model to the total number of transitions to class 5 from either class 2 or class 4 before absorption (referred as $\bar{M}$ hereafter; Fig. 4D). This can be computed by first calculating the mean total number of times the process is in each transient state before absorption, starting from class 1. We denote this number as $M(i)$, $i \in$ (transient states). There are two absorbing states when $\beta = 0$, so the total number of transient states is $l =$ (total number of states) $- 2$. $M(i)$ is obtained from the so-called fundamental matrix N defined as $N = (I - Q)^{-1}$, where I is the $l \times l$ identity matrix and Q is the $l \times l$ submatrix of P (the transition probability matrix):

$$P = \begin{pmatrix} Q & R \\ O & S \end{pmatrix}.$$

Q defines the transition within the transient states. O is a $2 \times l$ matrix with all 0's; R concerns the transtion from transient to absorbing states; S is the 2×2 identity matrix in our model. The first row of N corresponds to $M(i)$. The formula for fundamental matrices (and also for the mean and variances of absorption time) is according to Kemeny and Snell [6]. Computing the mean of $\bar{M}$, $\langle \bar{M} \rangle$, is straightforward using $M(i)$:

$$\langle \bar{M} \rangle = \sum_i \sum_j M(i)\, p_{i,j},$$

where $i \in$ (class2 $\vee$ class4), $j \in$ class 5 and $p_{i,j}$ is the transition probability from state i to j. Note that, when $\alpha = 0, \bar{M} = 1$ for all $n \geq 1$ because once in class 5 the process never leaves the class.

References

[1] Norris, J. R. *Markov chains* (Cambridge University Press, Cambridge, UK, 1998), 1st pbk. ed edn.

[2] Tanaka, T. U. Kinetochore-microtubule interactions: steps towards bi-orientation. *EMBO J* **29**, 4070–82 (2010).

[3] Nicklas, R. B. & Koch, C. A. Chromosome micromanipulation. 3. spindle fiber tension and the reorientation of mal-oriented chromosomes. *J Cell Biol* **43**, 40–50 (1969).

[4] Kitajima, T. S., Ohsugi, M. & Ellenberg, J. Complete kinetochore tracking reveals error-prone homologous chromosome biorientation in mammalian oocytes. *Cell* **146**, 568–81 (2011).

[5] Bertsekas, D. & Tsitsiklis, J. *Introduction to Probability* (Athena Scientific, Nashua, NH, 2008), ed. 2 edn.

[6] Kemeny, J. G. & Snell, J. L. *Finite markov chains*. The University series in undergraduate mathematics (Van Nostrand, Princeton, N.J., 1960).

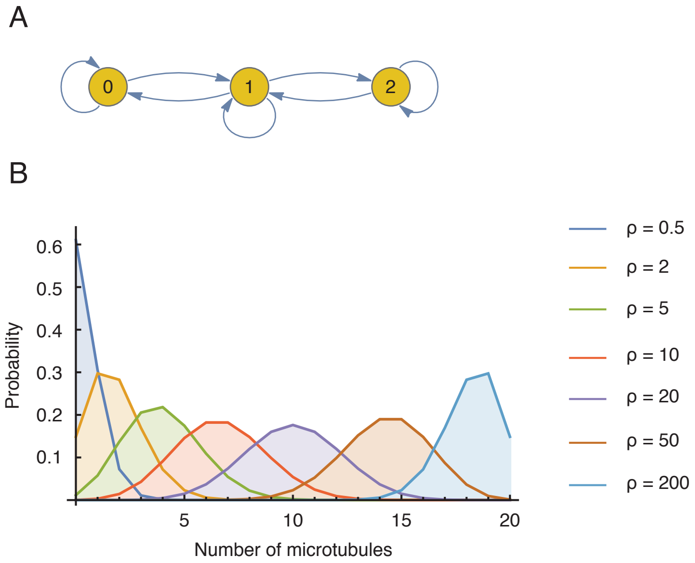

Fig. S1. A basic Markov chain model of kinetochore-microtubule interactions. (A) The Markov chain for $n = 2$. (B) Probability distribution of the number of kMTs (u_k) for $n = 20$ and ρ as indicated. See Additional file 1 for details.

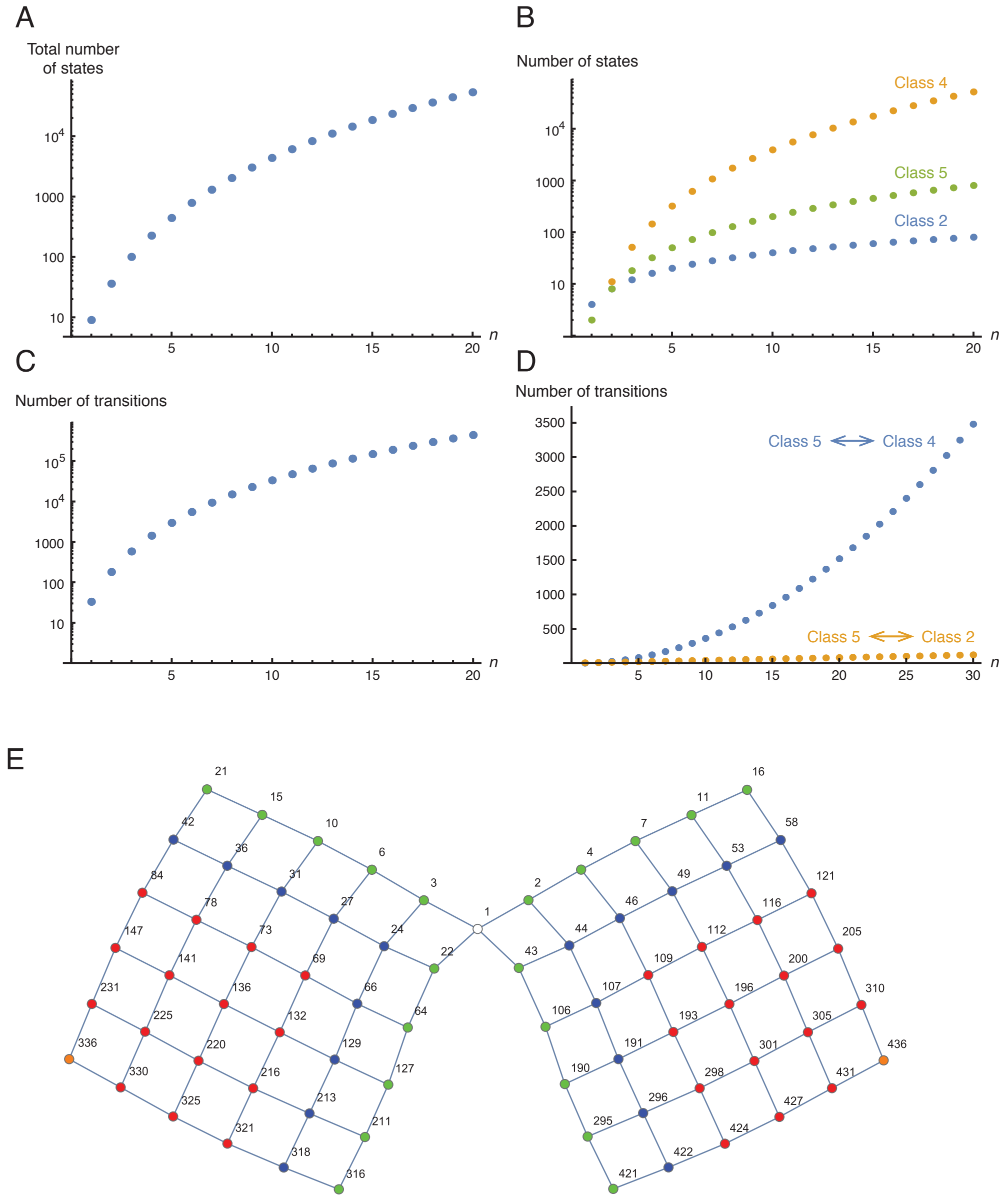

Fig. S2. The size and structure of the Markov chain in the full model (Model III).
For details of (A-D), see Additional file 1. (A) Total number of states. (B) Number of states in each class. Note that Class 3 and 5 have the same number of states. (C) Number of possible transitions. (D) Number of possible transitions in and out of class 5. (E) Graph of class 1, 2 and 5 for $n = 5$. All edges are bi-directional. Numbers indicate the indices of the states. Class 3 and class 4 are omitted. Class 5 (blue, red and orange) consists of two sub-classes corresponding to $r_n(i_1, 0, 0, j_2)$ and $r_n(0, j_1, i_2, 0)$. Two class 5 states (orange) correspond to those with max number of microtubules, i.e. $r_n(0, n, n, 0)$ and $r_n(n, 0, 0, n)$. Class 2 states are marked in green. Class 5 states can communicate with both class 2 and class 4. Note that every state in class 5 (red and blue) but two (orange) directly communicates with class 4. In contrast, only a minor fraction of class 5 (blue) can communicate directly to class 2, which are at the 'periphery' of the class 5 chain. Note that the amphitelic states in blue has $i = 1$ and/or $j = 1$, i.e. either of the kinetochores has only a single microtubule attached. As n grows, so do the two square lattices of the chain. But this graph structure remains the same.

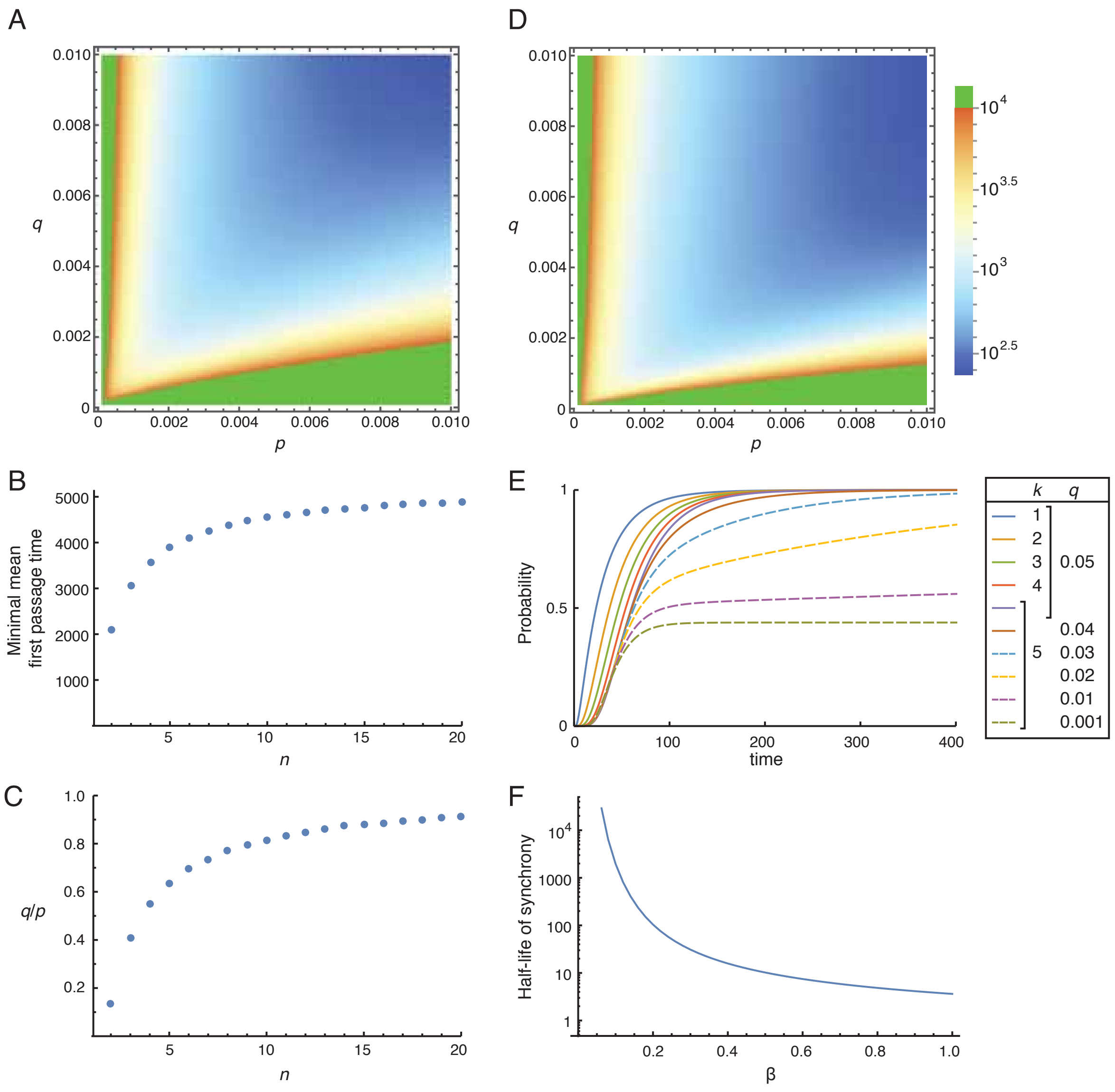

Fig. S3. Chromosome bi-orientation in meiosis and mitosis. (A) A density plot of mean first passage time to class 5 in meiosis I. $n = 10$. Green indicates >10,000. (B, C) The minimum mean first passage time in meiosis I (B) and the corresponding q/p ratio (C) for a fixed value of q ($= 0.0005$) plotted as a function of n. q/p approaches 1 while the minimum first passage time plateaus as n grows. (D) A density plot of the mean first passage time in mitosis. (E) Probabilities of synchrony in mitosis over time. k = number of pairs of sister chromatids, $p = 0.05$, $\beta = 0$. (F) Half-life of synchrony at steady states in mitosis. $k = 5$, $p = q = 0.05$. β needs to be small to maintain synchrony. In (D, E, F), $n = 10$, $\alpha = 0$ and $\gamma = 0.1$.

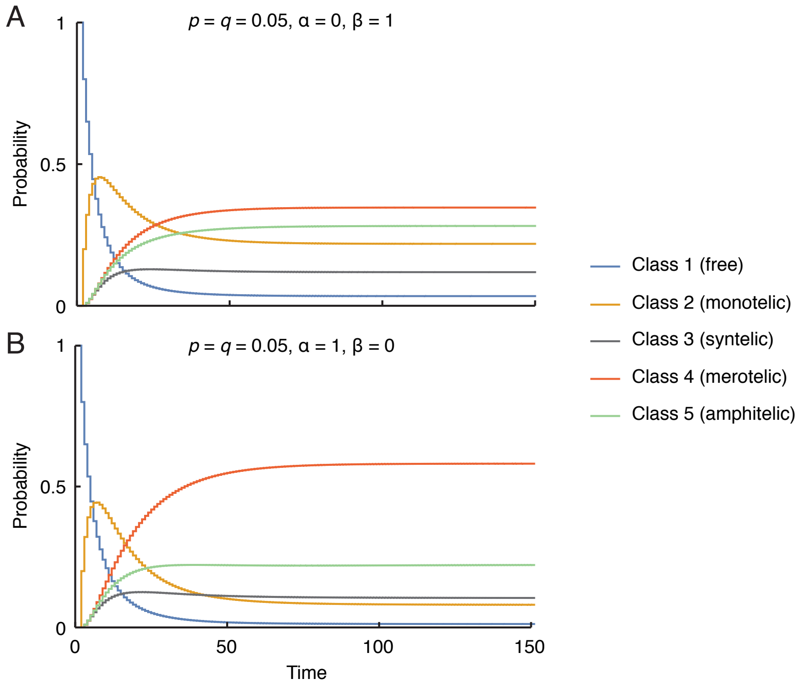

Fig. S4. Probability change of each class over time in meiosis I. (A) $\alpha = 0, \beta = 1$. (B) $\alpha = 1, \beta = 0$. The other parameter values are $n = 10, p = q = 0.05$.

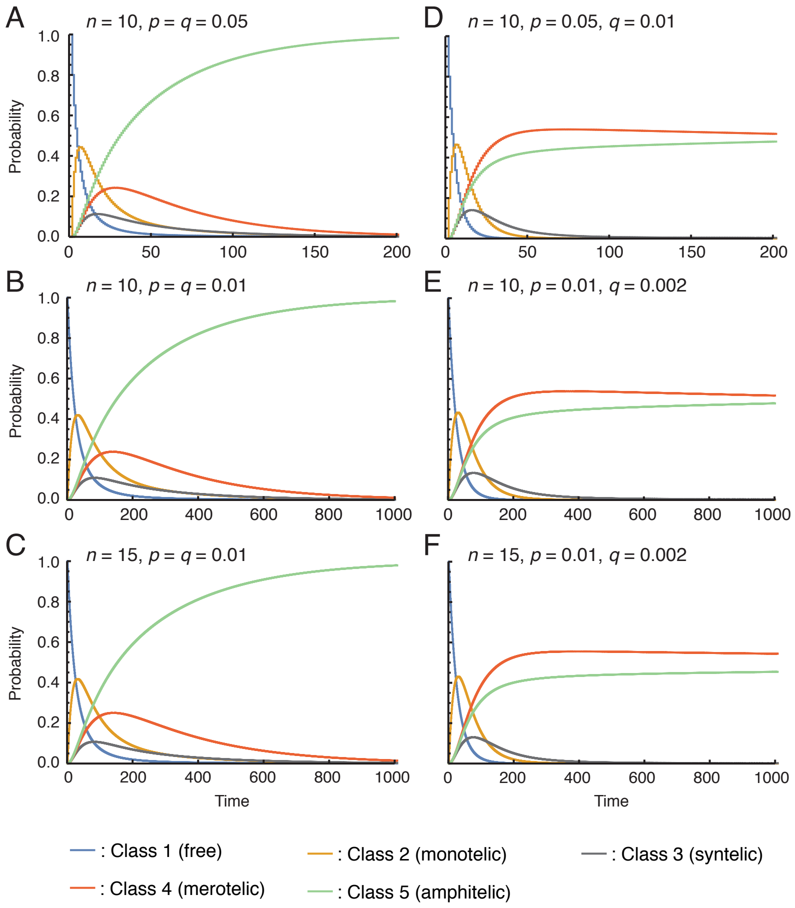

Fig. S5. Invariant dynamics of the Markov process with a constant *q*/*p* ratio. Examples of PMF time series (meiosis I) are shown. $\alpha = \beta = 0$ for all panels. Other parameters are as indicated. The dynamics are almost indistinguishable for a given *q*/*p* ratio besides the difference in time scale. Even with different *n*, they are qualitatively very similar (B versus C, or E versus F). See SI for details.

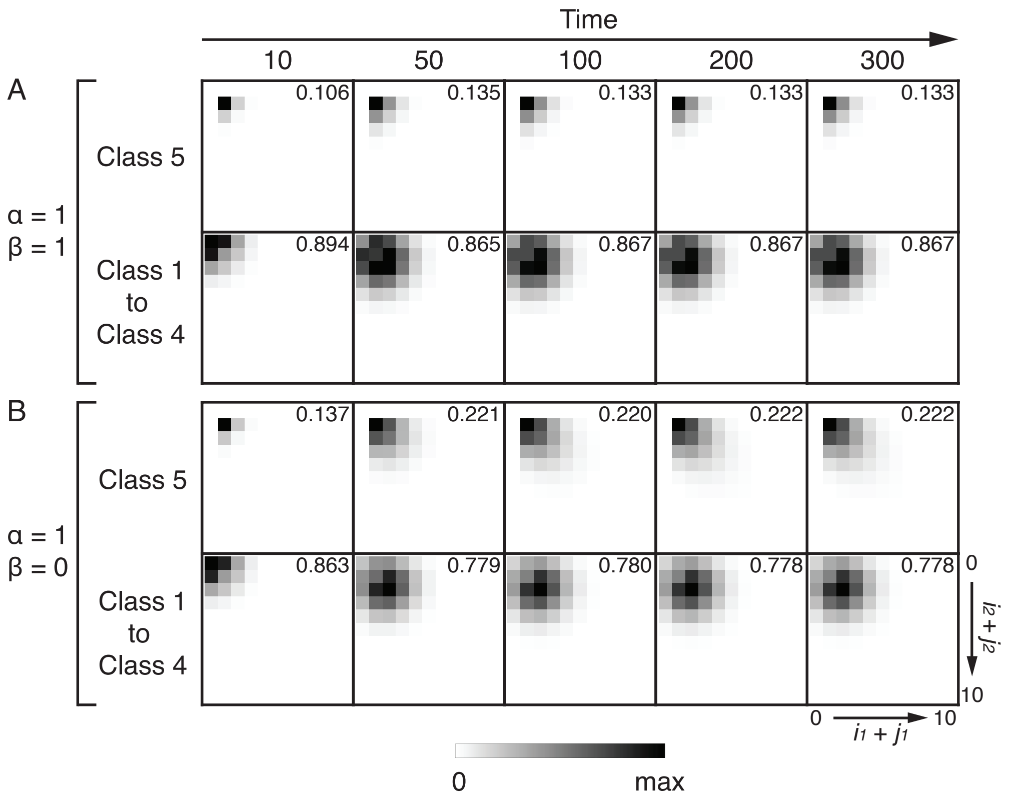

Fig. S6. Probability distribution of the number of kMTs over time in meiosis I.
The probability distributions of the kMT number are shown as density plots. Total probability of class 5 and class 1 to 4 are indicated on each panel. Parameters: $n = 10$, $p = q = 0.05$; α, β values are indicated on the left. kMT number distribution remains low (unstable) with α = 1.

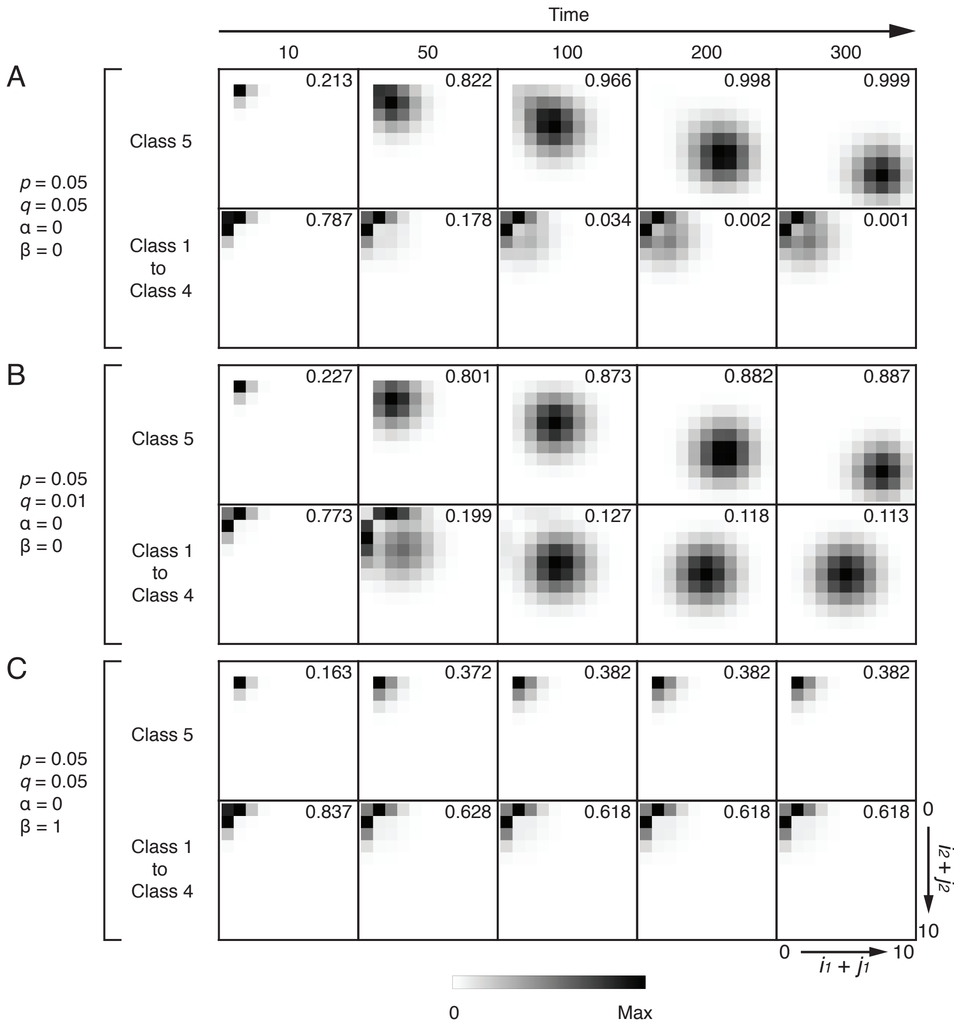

Fig. S7. Probability distribution of the number of kMTs over time in mitosis. Probability density plots of kMT numbers in 2D ($i_1 + j_1$ vs. $i_2 + j_2$; see Fig. 1C) at the indicated time points. Parameters are indicated on the left for each panel; $n = 10$ and $\gamma = 0.1$ for all panels. Probabilities are decomposed into class 5 and the rest (class 1 to 4) at each time point, Total probabilities are indicated on each panel. The densities are scaled from 0 to the maximal in each panel.

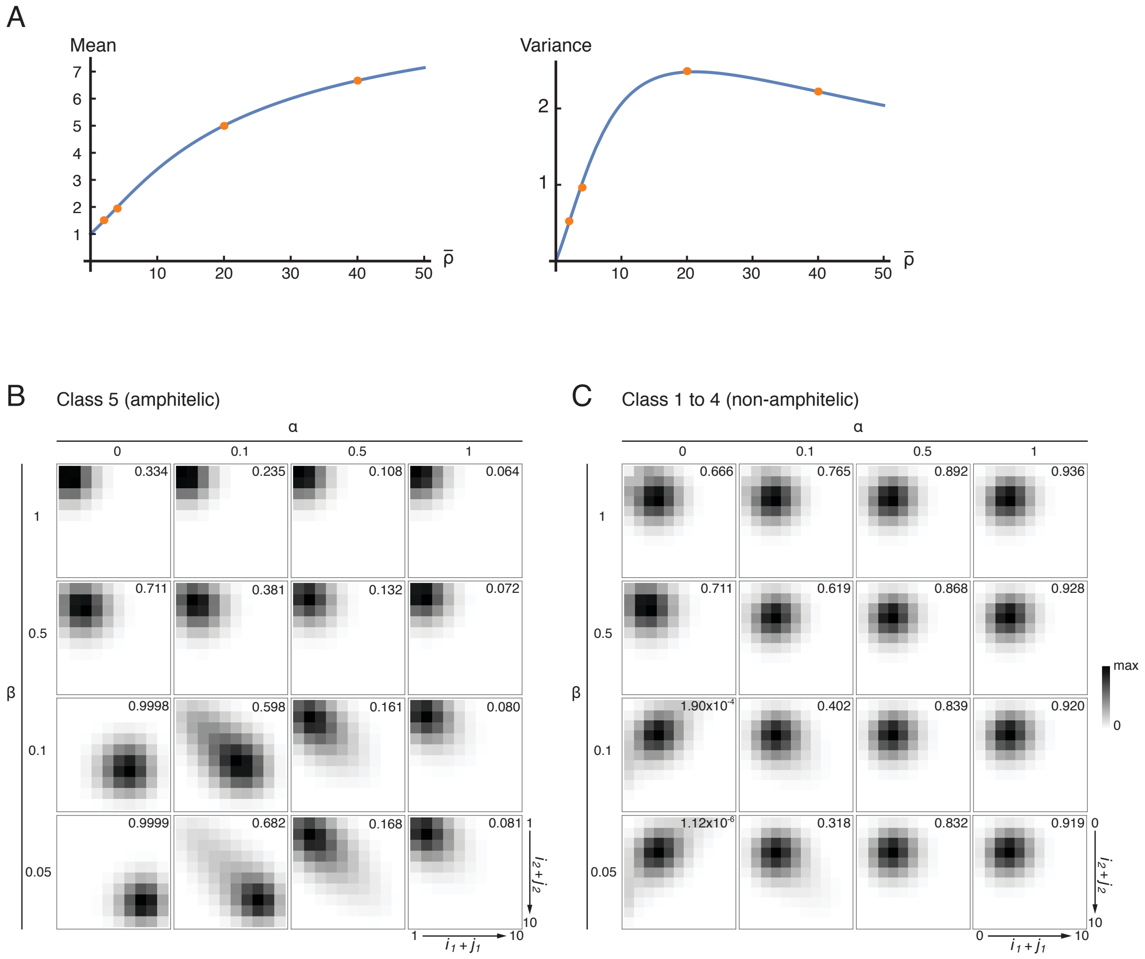

Fig. S8. Distribution of the number of kMTs at steady states. (A) An analytical approximation of kMT number in class 5 when $\alpha = 0$ at steady states. Orange indicates the sample points of the exact kMT numbers (mean and variance) derived from the steady-state PMF and blue curves the functions of approximation according to Eqs. (3) and (10). See Additional file 1 for the derivation of the approximation. Parameters: $n = 10$, $p = 0.05$, $q = 0.05$. (B, C) The probability distributions of kMT number in meiosis I with the indicated parameters α and β are shown as density plots. Total probabilities of class 5 (B) and class 1 to 4 (C) are indicated on each panel. Parameters: $n = 10$, $p = 0.1$, $q = 0.05$.

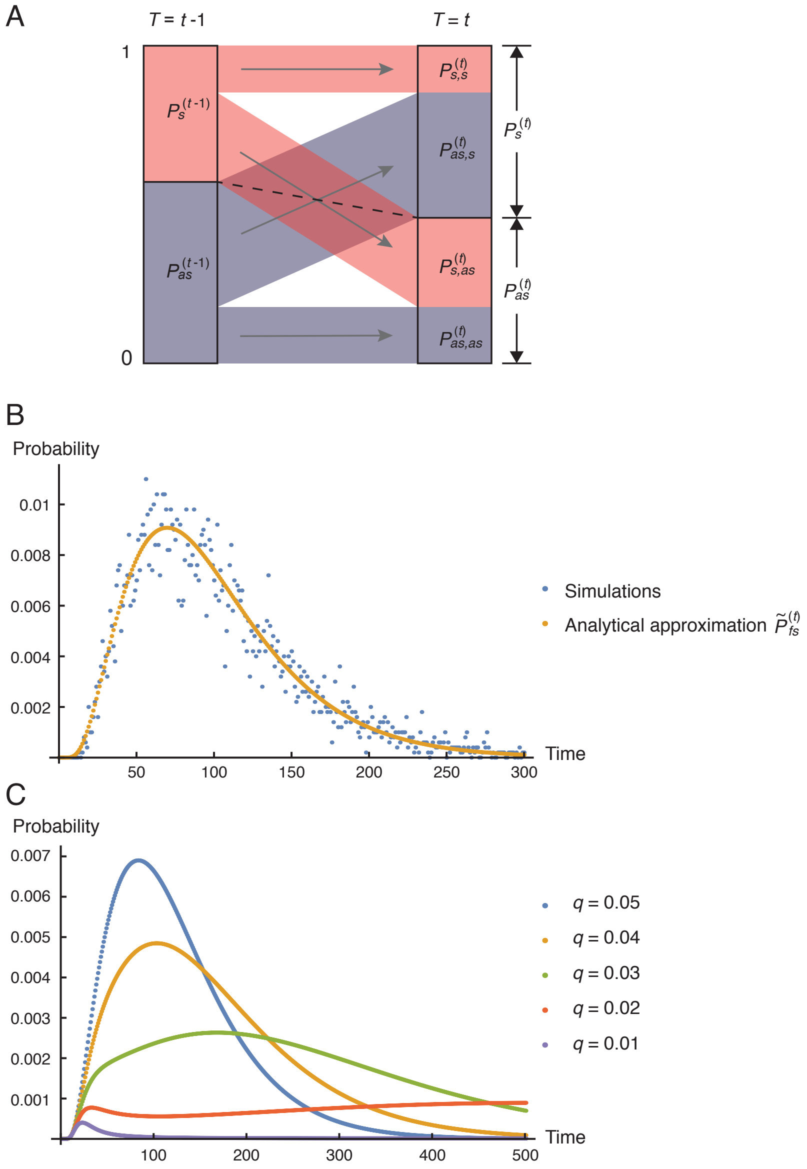

Fig. S9. Approximation of the probability of first synchrony. (A) Diagram of probability change of synchrony and asynchrony in each time step. For details, see Additional file 1 section 'Probability of synchrony'. (B) Analytical approximation of the probability of first synchrony (yellow) together with Monte Carlo simulation results (blue dots; probability at each time point in 5,000 simulations) is shown. Parameters: $n = 5$, $p = q = 0.05$, $\alpha = \beta = 0.01$. (C) Timing of the first synchrony with reducing q/p ratio. The plot shows the probability of the first synchrony at every time points in different conditions as indicated. As q/p ratio declines, the timing of first synchrony spreads more and more over time, becoming unpredictable. Parameter values: $n = 10$, $p = 0.05$, $\alpha = \beta = 0.05$ and number of bivalents (Markov processes) $k = 5$; $\gamma = 1$ (meiosis I).

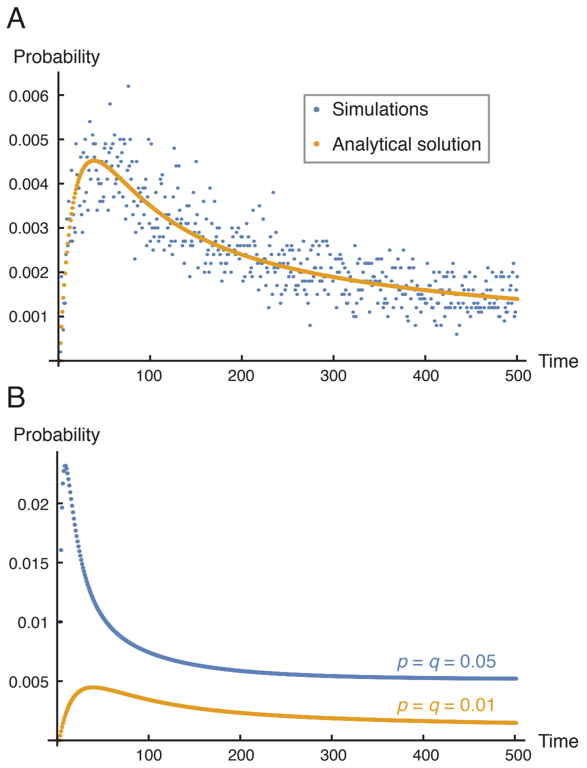

Fig. S10. Probability of bi-orientation attempts. (A) An analytical solution (yellow) and the probabilities of bi-orientation attempts obtained from simulations (blue dots; probability at each time point in 10,000 simulations) is shown. Parameters: $n = 5$, $p = q = 0.01$, $\alpha = \beta = 0.1$. This demonstrates a good fit of the analytical solution to the data obtained by simulations.
(B) Probability time series of bi-orientation attempts. Parameters: $n = 10$, $\alpha = \beta = 0.1$. Bi-orientation attemps are more frequent with $p = q = 0.05$ than with $p = q = 0.01$. The probability of bi-orientation attempts at steady states (at any time point) decreases from ~0.033 with $p = q = 0.05$ to ~0.0066 with $p = q = 0.01$. See Additional file 1 for the derivation of the analytical solution.
